# The potential of tailed amplicons for SARS-CoV-2 detection in Nucleic Acid Lateral Flow Assays

**DOI:** 10.1371/journal.pone.0301234

**Published:** 2024-05-10

**Authors:** João M. Vindeirinho, Ricardo Oliveira, Eva Pinho, Raquel Guiomar, Nuno F. Azevedo, Carina Almeida

**Affiliations:** 1 I.P–National Institute for Agrarian and Veterinarian Research, INIAV, Rua dos Lagidos, Lugar da Madalena, Vairão, Portugal; 2 Faculty of Engineering, LEPABE–Laboratory for Process Engineering, Environment, Biotechnology and Energy, University of Porto, Porto, Portugal; 3 Faculty of Engineering, ALiCE–Associate Laboratory in Chemical Engineering, University of Porto, Rua Dr. Roberto Frias, Porto, Portugal; 4 INSA, I.P–National Institute of Health Dr. Ricardo Jorge, Lisboa, Portugal; 5 Center of Biological Engineering (CEB), University of Minho, Braga, Portugal; University of Houston, UNITED STATES

## Abstract

Nucleic Acid Lateral Flow Assays (NALFAs) are a promising solution for the point-of-care detection of viruses like SARS-CoV-2. However, they show some drawbacks, such as the great dependency on the use of antibodies and the need for post-amplification protocols that enable the preparation of amplicons for effective readings, as well as low sensitivity. Here, we developed amplicons of a specific SARS-CoV-2 gene tailed with single-strand DNA (ssDNA) sequences to hybridize with DNA probes immobilized on the NALFA strips, thus overcoming the aforementioned problems. Results have shown that tailed primers have not compromised the amplification efficiency and allowed the correct detection of the amplicons in the lateral flow strip. This approach has presented a limit of detection (LOD) of 25 RNA copies /reaction mix (1 copy/μL) and the test of cross-reactivity with other related viruses has not shown any cross-reactivity. Twenty clinical samples were evaluated by NALFA and simultaneously compared with the gold standard RT-qPCR protocol, originating equal results. Although the number of clinical specimens tested being relatively small, this indicates a sensitivity and specificity both of 100%. In short, an alternative NALFA was successfully implemented, rendering an accurate route for SARS-CoV-2 diagnosis, compatible with low-resource settings.

## 1. Introduction

Vaccines and the fast and generalized testing have been the main technological breakthroughs in managing the public health crisis caused by COVID-19 [1]. The tools used for the detection of SARS-CoV-2 range from protein-based antigen tests, widespread due to their point-of-care test (POCTs) format, to the nucleic acid amplification tests (NAATs), which can be carried out either at clinical settings or as POCTs [2]. Solutions recurring to laboratory-based NAATs are well established, with standardized procedures. The NAAT-based POCTs have not achieved the same level of implementation, mostly due to a lack of technological maturity [3]. However, the testing capacity of clinical facilities was insufficient to tackle with the great demand for laboratory based-NAATS during the height of the pandemic, urging the creation of reliable POCTs, or even simpler versions of traditional NAATs to reduce the burden put on clinical laboratories [3,4].

Reverse transcription–real-time PCR (RT-qPCR), the dominant technique for the detection of SARS-CoV-2, requires costly qPCR instruments. On the other hand, end-point detection after PCR amplification, usually based on agarose gel electrophoresis, demands a significant amount of time and skilled human resources, which is not compatible with rapid diagnosis [5].

The Nucleic Acid Lateral Flow Assay (NALFA) is a cheap, fast, and easy-to-use end-point detection platform that offers a simple visual readout. It comprises the detection of amplicons (amplification products), or its ssDNA (single-stranded DNA) derivates, in a test strip. These molecules are submitted to a unidirectional flow in a liquid medium that promotes their interaction with capture molecules immobilized on a membrane, which is part of test strip [3]. Colorimetry is the most popular physicochemical detection approach applied in NALFA, usually using gold nanoparticles (AuNPs) as reporter molecules [6]. AuNPs are generally attached to 1) antibodies targeting specific antigens on the extremities of amplicons, or 2) oligonucleotide probes that hybridize with ssDNA amplicon´ products [3]. In order to perform the anchoring of the amplicons attached to the AuNPs on the test zone(s) of the strip, the amplicons are often labeled with biotin at the 5´end, by biotinylated primers during the amplification step, taking advantage of the interaction with streptavidin, directly adsorved on the strip [7,8].

The use of antibodies in the molecular mechanism of a NALFA has some disadvantages that may influence its accuracy to a greater or lesser extent [8–10]. The heterogeneity within the antibody pool, in the case of polyclonal antibodies may originate non-specific interaction with the antigen, which menaces test reproducibility and increases the chance of cross reaction (resulting in false positives), or variable signal intensity (resulting in false negatives) [11]. Despite monoclonal antibodies being an alternative to this heterogeneity, these aren´t always the option [11]. There is also a need for intricate molecular assemblies, often with various pairs of antibody-antigen, which introduces growing entropy, as the number of test lines increases [9]. Furthermore, the production of ssDNA products that could hybridize with oligonucleotide probes attached on the strip often demand a post-amplification processing step (by using CRISPR, or specific enzymatic treatments) that adds time and optimization needs to the assay [8,9,12].

The current work aimed to develop and validate a simple, accurate and elegant solution for end-point detection of nucleic acids, in the context of SARS-CoV-2 diagnosis, which would rely only on the interaction of complementary ssDNA. For this purpose, ssDNA tails were added to the amplicons allowing their simultaneous binding to the oligonucleotide-coated AuNPs and capture by oligonucleotide probes immobilized on the test strip. By using this NALFA approach, the use of antibodies for the detection on the strip was avoided, as well as the use of post-amplification protocols to enable the preparation of amplicons for effective readings, while resorting to a simple strategy based on the hybridization of ssDNA molecules. PCR, a simple and robust technique, was adopted for the enrichment of the samples and production of the desired tailed amplicons, thus allowing the direct detection on the test strip.

## 2. Experimental procedures

### 2.1. Oligonucleotide sequences

The primers and probes targeting E and RdRp genes (Table 1), used in the PCR amplification reactions, were taken from the protocol developed by Charité-Universitatmedizin, Berlin [13]. The ssDNA tails used to modify the primers, were adapted from existing literature [8]. The probes used to assemble the NALFA were also adapted from existing literature [8] (Table 1). The sequences of the tailed primers and oligonucleotide probes were analyzed using Oligoanalyzer (IDT) for simulating the probability of forming hetero- and homodimers. The capture test probe hybridizes with the tail of the forward primer. The ssDNA of the reporter probe is complementary to the tail of the reverse primer and the capture control probe. The primers, PCR oligonucleotide probe, capture and control probes were ordered from IDT, (United States), or Eurofins, (Luxembourg,), while the reporter probe attached to AuNP by a polymer bridge (ssDNA-AuNP), using an intellectually protected method was purchased from Nanopartz (United States).

**Table 1:** List of primers and probes used for E and RdRp genes amplification, as well as the probes used in the NALFA device, for the recognition of amplicons of both genes. The PCR probe was modified with 6-Carboxyfluorescein (FAM) and BlackBerry^TM^ Quencher (BBQ). The capture probes were modified with biotin and triethylene glycol (TEG); the reporter probe was modified with a thiol group (THIOL), in order to attach AuNP.

	Oligonucleotide type	Sequences (5´-3´)
E	Forward primer	ACAGGTACGTTAATAGTTAATAGCGT
	Reverse primer	ATATTGCAGCAGTACGCACACA
	Tailed forward primer	GTTTTCCAGTCACGAC-C3-ACAGGTACGTTAATAGTTAATAGCGT
	Tailed reverse primer	TGTAAAACGACGGCCAGT-C3-ATATTGCAGTACGCACACA
	PCR Probe	FAM-ACACTAGCCATCCTTACTGCGCTTCG-BBQ
	Capture test probe	GTCGTGACTGGGAAAACTTTTTTTTTTTTTTT-BIOTIN-TEG
	Capture control probe	TGTAAAACGACGGCCAGTTTTTTTTTTTTTTTT-BIOTIN-TEG
	Reporter probe	ACTGGCCGTCGTTTTACATTTTTTTTTTTTTTT-C3-THIOL-AuNP
RdRp	Forward primer	GTGARATGGTCATGTGTGGCGG
	Reverse primer	CARATGTTAAASACACTATTAGCATA
	Tailed forward primer	ATT ACG ACG AAC TCA ATG AA -C3- GTGARATGGTCATGTGTGGCGG
	Tailed reverse primer	CTA AGT AGC CGA ATT CCT AG -C3- CARATGTTAAASACACTATTAGCATA
	PCR probe	FAM-CAGGTGGAACCTCATCAGGAGATGC-BBQ
	Capture test probe	TTCATTGAGTTCGTCGTAATTTTTTTTTTTTTTTT-Biotin-TEG
	Capture control probe	CTA AGT AGC CGA ATT CCT AG TTTTTTTTTTTTTTT-BIOTIN-TEG
	Reporter probe	CTA GGA ATT CGG CTA CTT AGT TTTTTTTTTTTTTTT-C3-THIOL-AuNP

### 2.2. Nucleic acid amplification

The mastermix used for qPCR /PCR was NZY Supreme qPCR Probe Master Mix (2X), and for RT-qPCR /RT-PCR was NZY Supreme One-step RT-qPCR Probe Master Mix (2X). Primers/probes concentration, as well as PCR conditions have also followed the protocol developed by Charité-Universitatmedizin (Corman et al., 2020). Primers specific for E gene were added at a concentration of 0.4 μM (both forward and reverse), and 0.6μM of primer forward and 0.8μM of primer reverse for the RdRp gene. The oligonucleotide probe was added at a concentration of 0.2 μM for both genes [13]. Assays were run in the CFX96 Touch Real-Time Detection System using the same conditions for qPCR (PCR) and RT-qPCR (RT-PCR): two minutes at 95°C, 45 cycles with 5 seconds at 95°C and 30 seconds at 60°C. The total volume of the qPCR /PCR reaction was 25μL. The EDX SARS-CoV-2 standard (Bio-rad Laboratories, United States), containing 200 copies/μL, was adopted as RNA standard. A plasmid DNA solution (Eurofins, Luxembourg), containing 3X10^8^ copies/μL was used as DNA standard.

To estimate the primers efficiency, serial dilutions of the DNA standard, (500000, 50000, 5000, 500, 50, 5 number of copies per 25μL) were prepared and the amplification efficiency was determined. The quantification cycle (Cq) values, obtained using the Biorad RT-qPCR software (Bio-Rad CFX Manager IDE), were plotted as a function of the DNA copy number. Then the slope of the graph was used to calculate the efficiency of amplification, according to Eq 1 [14].


$$Amplification\;efficency\;(\text{\%})=\left[10^{\left(\frac{-1}{\;slope}\right)}-1\right]\times 100$$ (1)

### 2.3. NALFA assembly

The components of the strip were all manually assembled. These included the sample pad (1.5 cm, glass fiber membrane, grade 8950 (Ahlstrom Munksjo, Finland)); the test zone (2.5 cm, nitrocellulose membrane (FF170HP, Whatman plcUnited Kingdom)) and the absorbent pad (2 cm, absorbent paper grade (270, Ahlstrom Munksjo, Finland)), all supported by a backing card (KN-2211, Kenosha, Netherlands) with 5 mm width. The capture probes’ immobilization on the strips was performed based on the anchor system biotin-streptavidin. A streptavidin solution (5mg/mL, Alfa Aesar, United States) and biotinylated ssDNA capture probes (1.5mg/mL) were conjugated in PBS, ensuring a 1:4 streptavidin:biotin ratio. Then, test and capture probes, at a concentration of 1.5mg/mL were added to the strip, while pipetting 0.5ul and allowing air-drying for one hour.

### 2.4. NALFA optimization

Five running buffers were evaluated to establish an efficient flow in the strip. Selected buffers included (1) modified saline-sodium citrate buffer (M-SSC, 0.15 M sodium citrate, 1.5 M NaCl, 3.5% v/v triton X-100, 0.25% v/v SDS and 12.5% v/v formamide, pH 7.0); (2) phosphate buffered saline (PBS, 140 mM NaCl, 2.7 mM KCl, 10.1 mM Na_2_HPO_4_, 1.8 μM KH_2_PO_4_, pH 7.0); (3) tris-buffered saline (TBS-T, 20 mM Tris-HCl, 130 mM NaCl, 0.05% w/v Tween 20, pH 7.5); (4) tris-EDTA-NaCl-Tween20-Casein (TENTC, 50 mM Tris, 1 mM EDTA, 0.15M NaCl, 0.05% w/v Tween 20, 2% w/v Casein, pH 8.0); and (5) boric buffer (100 mM HBO_3_, 0.05% w/v Tween 20, 1% w/v BSA, pH 7.5) [8,15].

The amplicons (10 μL) were added to 5 μL of AuNPs (with 15 nm or 40 nm) conjugated with a reporter probe and 10 μL of running buffer. The concentration of capture probes (1, 1.5 and 2 mg/mL) and the amount of AuNPs used (5μL with 7.53X10^12^,1.88X10^13^ and 3.77X10^13^ copies/mL, corresponding to optical densities (ODs) of 5, 12.5 and 25) was also optimized; assuring that AuNP are in excess in relation to the tailed primers/capture probes. The stock of AuNPs had a concentration of 7.53X10^13^ copies/mL, corresponding to an OD of 50. This mixture (25μL) was vortexed during 5 seconds and then incubated for 3 minutes at room temperature. The mixture was applied to the strip’ sample pad and the results were registered after 10 minutes.

### 2.5. Evaluation of NALFA detection limit and cross-reactivity

The Limit of Detection (LOD) of the method was determined by testing serial dilutions of the RNA standard (2000, 500, 50, 25, 10 and 5 number of copies per 25 μl of reaction mix). After amplification, applying the condition mentioned in section 2.2 for RT-qPCR, samples were loaded into the optimized NALFA strip. More precisely, 10 ul of the PCR reaction were mixed with 5 μL of AuNPs (40 nm) and 10 ul of buffer. The strip was then placed into the tube with the mixture and allowed to react for 10 minutes before reading the result. For evaluating the NALFA cross-reactivity (analytical specificity) a panel of 14 RNA viruses (including other coronavirus, influenza and other respiratory viruses) and 2 bacterial DNA samples were gathered and tested according to the procedures described in section 2.2 and 2.3, while using 5μL of viral sample.

### 2.6. Evaluation of NALFA with clinical samples

Twenty clinical samples, consisting of nasopharyngeal swabs obtained from patients suspected of SARS-CoV-2 infection (archived samples from the internal collection of the National Institute of Health Dr. Ricardo Jorge (INSA)) were used to validate the developed NALFA. The clinical samples were accessed in two independent assays. The RNA extraction of the clinical samples (200 μL) was performed with the High Pure Viral RNA Kit (Roche Molecular Systems, United States), following the kit instructions. Then, samples were tested using either the RT-qPCR Charité protocol [13], or the NALFA after tailed RT-PCR amplification as described above. The volume of RNA extracted from clinical samples added to each RT-qPCR, or RT-PCR run was 10μL.

## 3. Results

### 3.1. Amplification efficiency with tailed primers

**Fig 1:**
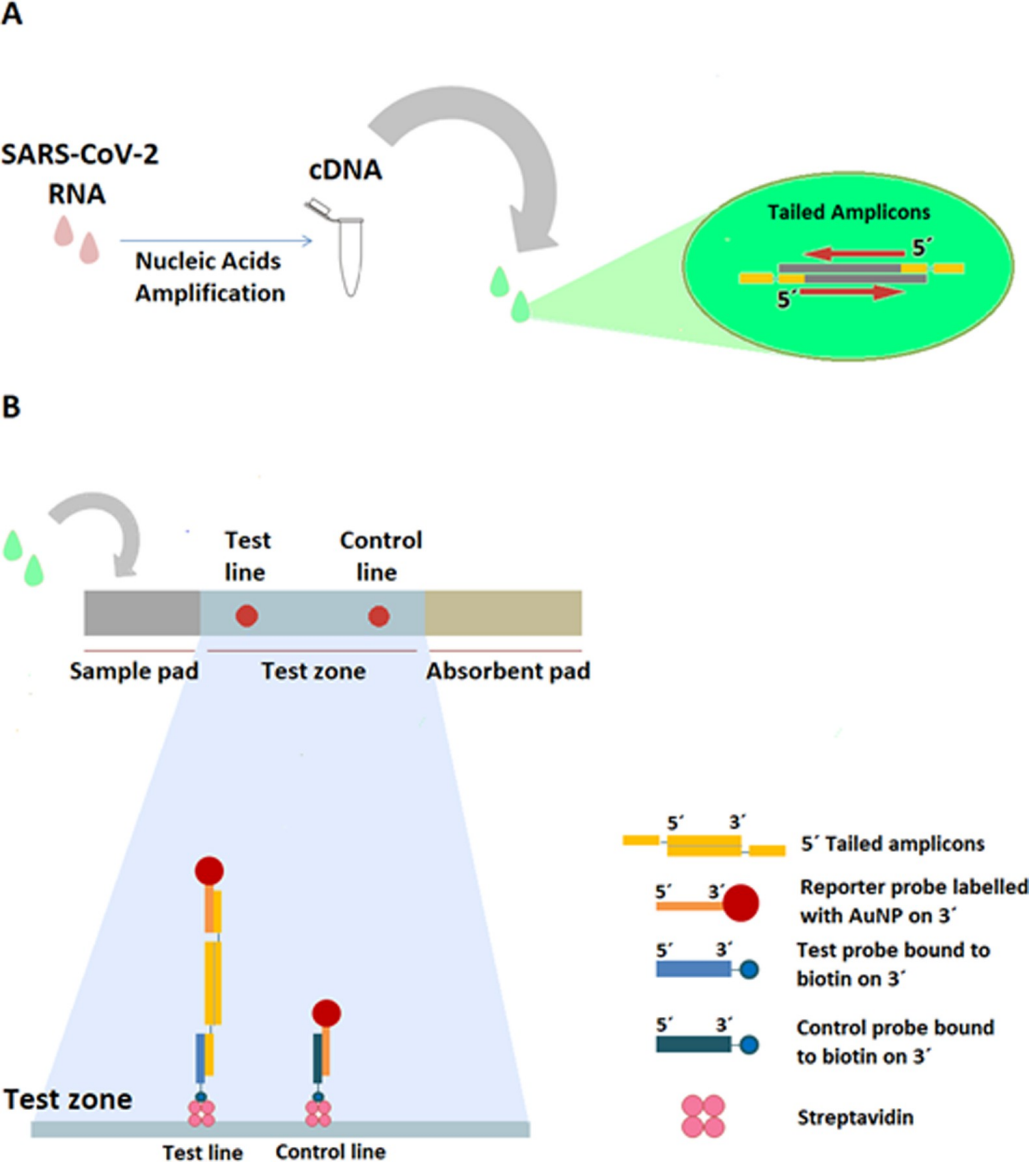
Schematic representation of the developed NALFA. (A) After the collection of the sample, the RNA is subject to reverse transcription, which occurs simultaneously with cDNA amplification. In this amplification process specific amplicons with ssDNA tails in the 5´ends are produced. (B) Detail of the reaction that occurs on the test and control zones of the NALFA. In the test line, the tailed amplicon (previously hybridized with the oligonucleotide reporter probe on the AuNP) and the oligonucleotide capture test probe hybridize; assuring specific recognition in the control line, the reporter probe interacts with the capture control probe that is complementary to the reporter probe attached to AuNP.

The present work aimed to develop a NALFA platform for cDNA detection, following RNA processing and amplification (Fig 1A). This approach is based on hybridization of ssDNA tails inserted into 5´ends of amplicons with specific oligonucleotide capture probes immobilized on the NALFA strip (Fig 1B) or specific reporter probes immobilized on the surface of the AuNPs. The capture probes were labelled with biotin in the 3´end, which enables its immobilization on the strip by recurring to the biotin-streptavidin interaction (Fig 1B).

The success of the developed NALFA is directly dependent on the amplification of the target gene using tailed primers. Therefore, the amplification´ efficiency of the genes E and RdRp using primers with and without tails was compared (S1 File). The tailed primers proved to be effective for the amplification of both genes, originating amplification profiles similar to those obtained with primers without tails (S1 File). The amplification efficiency of tailed primers for gene E was 83%, while the amplification efficiency of non-tailed primers was 89% (S1 File). Regarding gene RdRp, the amplification efficiency of tailed primer was 81% and 76% for non-tailed primers (S1 File).

### 3.2. Assembly and optimization of the NALFA

**Fig 2:**
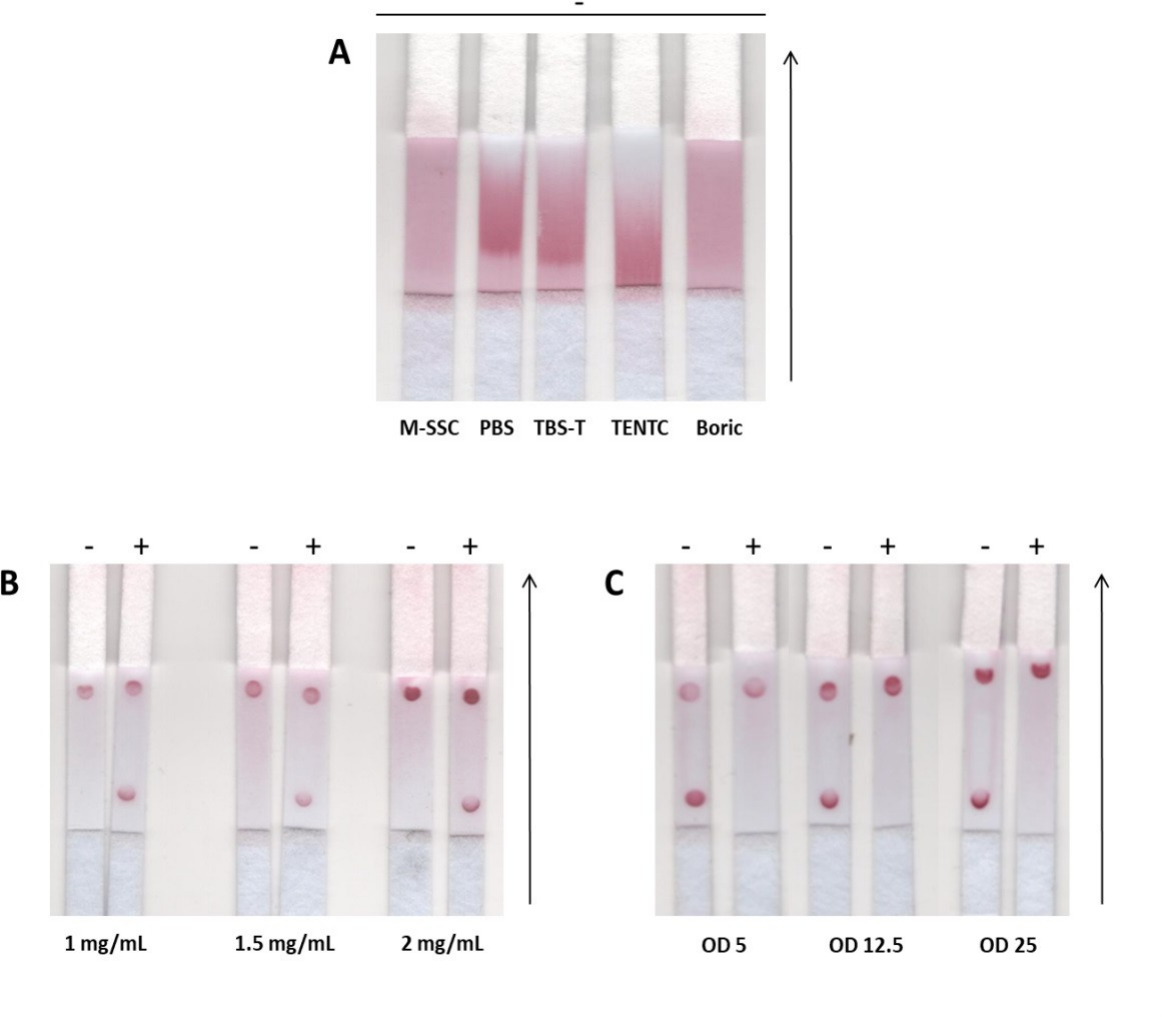
Optimization of factors influencing the detection efficiency of the NALFA, using E gene as a model. (A) Influence of the running buffer on the migration of AuNPs along the membrane materials; (B) Effect of the concentration of biotinylated capture control and test probes, expressed in mg/mL, in the colorimetric signal (red dots); and (C) effect of the ssDNA-AuNPs concentration, expressed in OD values in the signal intensity. The signal (+) represents positive samples; while the (-) represent negative samples. Arrows depict the samples running direction.

NALFA requires a strip (with a sample, test and absorbent zones), a running buffer, colorimetric reporter molecules and probes. The material used to construct each zone of the strip was defined according to the literature [8]. Regarding the running buffer, five buffers (M-SSC, PBS, TBS-T, TENTC, Boric), with distinct pH values, salts, blocking agents, or detergent composition were tested. The migration rate of the AuNPs (15 nm, functionalized with the reporter probe) in solution with each buffer was used as selection parameter. For the buffers PBS, TBS-T and TENTC, the AuNPs migration ended before the solution reached the absorbent pad. M-SSC proved to be as effective as Boric buffer (Fig 2A) in allowing an efficient flow of the tailed-AuNPs. The optimal concentration of the test and control capture probes was also assessed. As observed in Fig 2B, there were no great differences for the concentration tested; nonetheless, 1.5 mg/mL was selected as the probes concentration for further tests. The AuNPs added at a concentration of 1.88E+13 (OD 12.5) showed to be the best compromise between the concentration of nanoparticles added and the intensity of the spot (Fig 2C).

### 3.3. Determining the limit of detection (analytical sensitivity)

**Fig 3:**
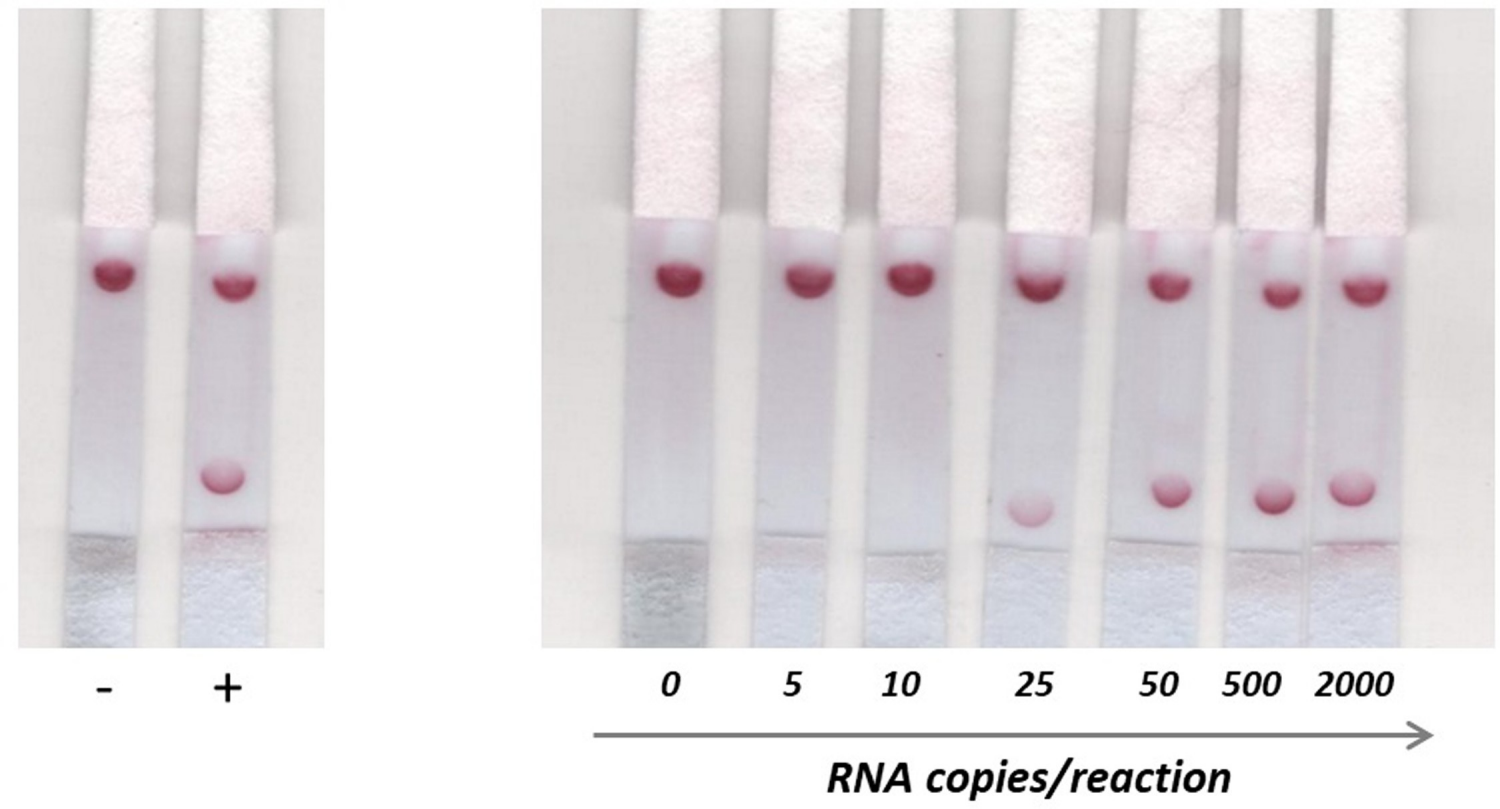
Determination of the limit of detection (LOD) of the developed NALFA, for RdRp gene. A range of initial RNA concentrations expressed as the total number of copies between 0 (NTC) and 2000 were used for RT-PCR amplification; being then used to assess the LOD of the developed NALFA.

The LOD of the NALFA system was evaluated for both genes, by testing on strips the amplicons generated by end-point RT-PCR, after simultaneous reverse transcription and DNA amplification. The lowest amount of RNA detected by the NALFA resulted from the amplification of 25 RNA copies/reaction (1copy/μL), when targeting both E and RdRp genes (Fig 3). Table 2 shows information on the reproducibility of the LOD.

**Table 2:** Results obtained for the LOD determination of the NALFA system for both genes E and RdRp. The amplicons generated by end-point RT-PCR for different/decreasing initial RNA concentrations were tested on strips and expressed by the number of positives out of 3 independent assays.

SARS-CoV-2 RNA Total copies	Gene E (Positives/Total number of assays)	Gene RdRp (Positives/ Total number of assays)
**2000**	(3/3)	(3/3)
**500**	(3/3)	(3/3)
**50**	(3/3)	(3/3)
**25**	(2/3)	(3/3)
**10**	(1/3)	(0/3)
**5**	(0/3)	(0/3)
**0**	(0/3)	(0/3)

Fig 3 shows the results for RdRp gene as an example, although the same was made for E, which is present in supplementary materials.

### 3.4. Determining the cross-reactivity with a panel of distinct microorganisms (analytical specificity)

The cross-reactivity of the assay was determined by screening a panel of 14 related and unrelated viruses and bacterial strains (Table 3) in the NALFA, following RT-PCR amplification. According to the results, only SARS-CoV-2 controls (whole genome and plasmid DNA) were detected, as depicted in Table 3. The NALFA images can be found in supplementary materials.

**Table 3:** Cross-reactivity assay, targeting a panel of distinct viruses and bacteria. RNA/DNA extracts were subjected to RT-PCR amplification and then applied to the NALFA system. The signal (+) represents positive result; while the (-) represent negative results. Two independent assays were performed for the test of each viral sample, with the result of each assay listed below.

List of viruses used in cross-reactivity test			
Microorganism type	Strain	Result ‐ E	Result ‐ RdRp
SARS-CoV-related	SARS-CoV-2	**+ (2/2)**	**+ (2/2)**
	SARS-CoV-1	**- (2/2)**	**- (2/2)**
other coronavirus	coronavirus 229E	**- (2/2)**	**- (2/2)**
	coronavirus NL63	**- (2/2)**	**- (2/2)**
	coronavirus OC43	**- (2/2)**	**- (2/2)**
	MERS-CoV	**- (2/2)**	**- (2/2)**
influenza	influenza A (H1N1)	**- (2/2)**	**- (2/2)**
	influenza A (H3N2)	**- (2/2)**	**- (2/2)**
	influenza A (H5N1)	**- (2/2)**	**- (2/2)**
	influenza B (Victoria)	**- (2/2)**	**- (2/2)**
	influenza B (Yamagata)	**- (2/2)**	**- (2/2)**
Other respiratory viruses	parainfluenza	**- (2/2)**	**- (2/2)**
	human rhinovirus A16	**- (2/2)**	**- (2/2)**
	human rhinovirus B5	**- (2/2)**	**- (2/2)**
Bacteria	*Escherichia coli*	**- (2/2)**	**- (2/2)**
	*Staphylococcus aureus*	***-* (2/2)**	**- (2/2)**

### 3.5. Validating the NALFA with clinical samples

After evaluating the method performance in terms of LOD, the system was challenged by clinical samples. For this, clinical samples with known results for the presence of the Covid-19 virus were used. The samples were previously subjected to the standard RT-qPCR as described on the Charité protocol and the Cq values were obtained as presented in Table 4.

**Table 4:** Evaluation of 20 clinical samples of nasopharyngeal swabs obtained from patients suspected of SARS-CoV-2 infection (internal collection of the National Institute of Health Dr. Ricardo Jorge (INSA)) by RT-qPCR and NALFA. NA: No Amplification; NT: Not test due to a shortage of sample volume. NALFA results are presented by identifying each positive sample. The signal (+) represents positive result; while the (-) represent negative results. Two independent assays were performed for the test of each clinical sample, with the results of the test listed below.

Sample	E		RdRp	
	RT-qPCR (cq)	NALFA	RT-qPCR (cq)	NALFA
**1**	36.21	+ (2/2)	39.51	+ (2/2)
**2**	41.50	+ (2/2)	35.73	+(2/2)
**3**	40.98	+ (2/2)	37.14	+(2/2)
**4**	41.83	+ (2/2)	39.86	+(2/2)
**5**	NA	- (2/2)	NA	-(2/2)
**6**	43.19	+(2/2)	NT	NT
**7**	39.77	+(2/2)	38.18	+(2/2)
**8**	38.12	+(2/2)	38.13	+(2/2)
**9**	35.30	+(2/2)	39.14	+(2/2)
**10**	NA	-(2/2)	NA	-(2/2)
**11**	35.07	+(2/2)	38.95	+(2/2)
**12**	35.62	+(2/2)	40.25	+(2/2)
**13**	34.91	+(2/2)	32.78	+(2/2)
**14**	NA	-(2/2)	NT	NT
**15**	NA	-(2/2)	NA	-(2/2)
**16**	40.99	+(2/2)	41.53	+(2/2)
**17**	NA	-(2/2)	NA	-(2/2)
**18**	NA	-(2/2)	NA	-(2/2)
**19**	41.68	+(2/2)	33.11	+(2/2)
**20**	NA	-(2/2)	NA	-(2/2)

The results obtained for the developed NALFA are in agreement with the RT-qPCR results for all clinical samples regarding both genes. For gene E, 13 clinical samples were confirmed to be positive, while 7 were found to be negative (S1 File). Regarding gene RdRp, 12 clinical samples were confirmed to be positive, while 6 were found to be negative (S1 File). Since the results of the test of clinical samples targeting E gene are similar to those for gene RdRp, the images corresponding to the test of E gene were included in supplementary materials. Overall, and despite the limited number of samples, we have obtained a specificity and sensitivity both of 100%, for E (Sensitivity 95% Confidence Interval (CI): 75.29%-100.00% and Specificity 95% CI: 75.29% -100.00%) and RdRp gene detection (Sensitivity 95% CI: 73.54–100% and Specificity 95% CI:54.07–100%).

## 4. Discussion

In recent years, a few works reported the use of ssDNA tails in the extremities of amplicons for simultaneously enabling their capture by nucleic acid probes immobilized on the membrane of NALFAs and their binding to reporter molecules such as AuNPs [8,10,16]. The work developed here aimed to adapt this strategy for the detection of SARS-CoV-2 (Fig 1). To the best of our knowledge, the use of this system for detection of complementary DNA, following reverse transcription of RNA was reported here for the first time.

The interactions between the tail and primer sequences are crucial for the production of tailed amplicons [17]. The insertion of 5´ssDNA tails in the extremities of amplicons can have a variable impact on the amplification efficiency, affecting the primers performance or even impairing amplification [18]. According with existing literature, the tails can improve the amplification performance, but can also cause the disruption of the primer annealing, or the formation of artifacts, thus originating negative effects on the primers´ amplification performance [17,18]. In this study, the tailed primers´ amplification efficiency (85%) was slightly lower when compared to the value obtained with non-tailed primers (89%), for the case of E gene. However, in the case of gene RdRp the amplification efficiency of tailed primers was higher (81%) than that of non-tailed primers (76%). The values of amplification efficiency for both pairs of tailed primers are close to each other, pointing to a relatively similar amplification performance.

The NALFA proposed here generates an optical signal from AuNPs (colored red) that are able to bind either to the tailed amplicons (test signal) or to the capture control probe immobilized on the nitrocellulose strip (control signal). To obtain the strongest signal, each interaction between the DNA/amplicon, or the AuNPs and capture probes must be optimized. Thus, a set of running buffers were tested taking in account their properties as stabilizers (PBS), blocking agents (TBS-T, TENTC), or the improved performance in similar protocols (boric, M-SSC) [8,15]. Although M-SSC and boric enabled a similar migration rate of AuNPs (Fig 2A), M-SSC was chosen due to the widely documented germicidal activity of citrate (its main component) with direct effects on a long shelf-life of the NALFA [8,19].

In order to determine the proper concentration of probes a few factors must be taken in account, such as the amplicon concentration (capture test probe must be in excess to ensure that all the DNA present on the sample is captured); the stoichiometry between biotinylated probes and streptavidin; or the conjugation route (here occurred on tube before immobilization of the conjugate on the strip) [8]. The obtained results (1.5 mg/mL for the capture probes) are in accordance with other published works [8,16].

AuNPs are, probably, the most common reporter particles used in NALFAs mainly due to their capacity to produce a very intense red signal. The particle size must be chosen to ensure equilibrium between the signal intensity and the migration rate. Larger particles can provide a stronger signal per binding event, but their flow through the nitrocellulose membrane is slower [20,21]. Thus, AuNPs with 15 nm were selected, since the dimension of the AuNPs has been associated with the migration rate on NALFA’ membrane. Smaller nanoparticles, usually, migrate faster contributing to a quicker resulting signal [21–23].

After optimizing the strips composition and running solutions, the method performance was evaluated using known concentrations of RNA. According with information in literature and data sheets of commercial devices, the LOD of the developed NALFA method (25 copies or 1 copy/μL) had similar values to those described for other strategies relying on NALFA with previous nucleic acid amplification, (~1–4 copies RNA/μL), for SARS-CoV-2 detection, [8,24]. The results of the test cross-reactivity with other viral and bacterial genomes show evidence of an optimal analytical specificity, since there wasn´t cross-reactivity with any tested sample. Also, promising were the results obtained in clinical samples simultaneously accessed with NALFA and the RT-qPCR Charité protocol, which revealed equal results when tested with both techniques [13]. All the samples revealed concordant results for both genes, which highlight the robustness of this NALFA-based system.

Taking in account the characteristics of the developed NALFA, which can be compared withDetect Covid-19 Test^TM^ and Visby Medical Respiratory Health Test^TM^, it was concluded that our assay match both in terms of analytical sensitivity (a similar LOD, although) and specificity (total absence of cross-reactivity with other strains) [24,25]. Detect Covid-19 Test^TM^ is the only isothermal nucleic acid amplification assay with a NALFA read-out approved by FDA and the more recent Visby Medical Respiratory Health Test^TM^ is a miniaturized PCR device, with an integrated end-point LFA colorimetric read-out, after the interpretation of the product ‘technical data sheets [24,25]. Both commercial kits are aimed to be performed at the point-of-care (at home), with Visby Medical Respiratory Health Test^TM^ doing it in less than 30 minutes, while Detect Covid-19^TM^ takes about one-hour. The method here proposed, comprising RT-PCR and NALFA read-out, is not yet integrated in a platform for point-of-care use, since the main goal of this work is to highlight the potential of the NALFA strategy above exposed to yield reliable detection of cDNA following a nucleic acid amplification step from a RNA template. Nevertheless, there is potential to adapt our method to such a platform, by either using a miniaturized PCR device, or a portable PCR instrument. The running time of our assay is around 1 hour and 20 minutes, due to the running time of the PCR of about one hour, plus the time for performing RNA extraction (about 10 minutes) and the time for executing the NALFA itself. While there is a high potential for decreasing the time to result if an isothermal amplification is adopted in a later stage; it is important to stress that these methodologies are not yet as disseminated as PCR, which can impose limitation in terms of access to reagents/materials and reproducibility across labs.

Moreover, this NALFA system avoid the use of antibodies which production is still costly [11] and doesn´t need extra steps that often are required to get the amplicons ready for analysis on the strip following amplification [8,26]. Finally, the test format here reported allow the multiplex/simultaneous hybridization of the different tailed amplicons with the capture and reporter probes, facilitating the construction of multi-targets, by comprising several test lines complementary to different tailed primers.

In addition, the molecular strategy here described could offer an alternative for a NALFA in which the biotin-streptavidin pair doesn´t need to be adopted, by using another type of molecules to immobilize the capture oligonucleotide probes [27,28]. In fact, other similar systems simply remove any intermediate immobilization system, by adsorbing the oligonucleotides directly in the membrane [27].

## 5. Conclusions

Overall, the NALFA approach presented in here is an accurate and simple end-point assay for SARS-CoV-2 diagnosis that relies only on the interaction of complementary ssDNA sequences. It presents an excellent performance in terms of LOD, specificity and sensitivity. Furthermore, the junction of this NALFA with an end-point PCR originates an inexpensive, but reliable diagnostic method able to be used in low-resource facilities. This strategy can be helpful in settings without qPCR or when they are not available due to great demand of samples, making room for the traditional end-point PCR to become an option. Ultimately, the NALFA here presented could be combined with other amplification techniques that allow the generation of tailed amplicons, which creates enthusiasm about the development of easily deployable and reliable POCTs, adopting the referred system.

## Supporting information

S1 File Supporting information file containing all additional data mentioned in the manuscript text. (DOCX)
