# Supplementary material for: The potential of tailed amplicons for SARS-CoV-2 detection in Nucleic Acid Lateral Flow Assays
Source: PLoS One. 2024 May 10;19(5):e0301234. doi: 10.1371/journal.pone.0301234 (PMC11086916; doi:10.1371/journal.pone.0301234)
Supplement: S1 File — (DOCX) [file pone.0301234.s001.docx]

**Supplementary Material**

**
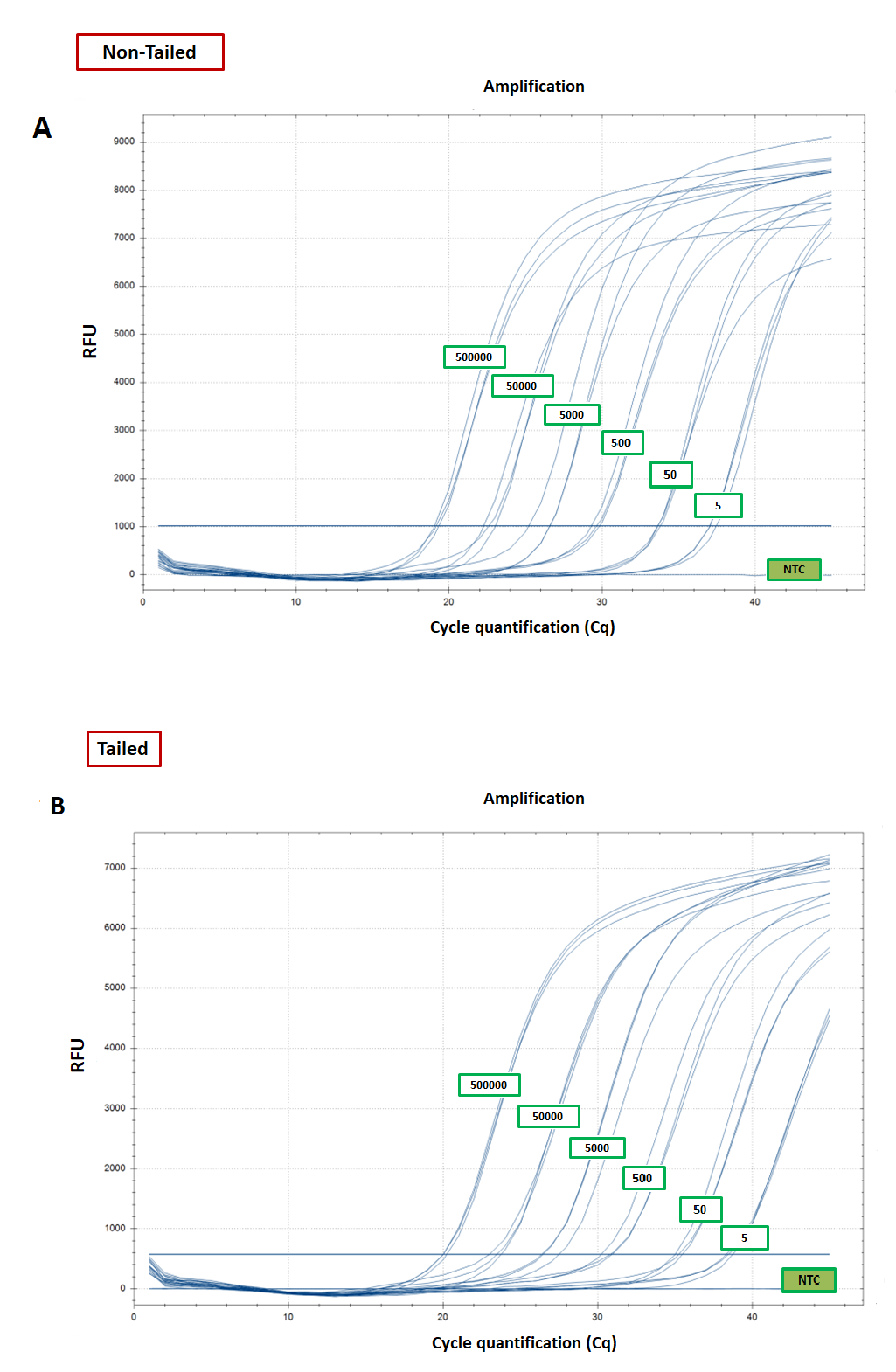
**

**S1 Fig.** Amplification profiles of E gene primers, when amplification is performed with A) non-tailed and tailed B) primers.


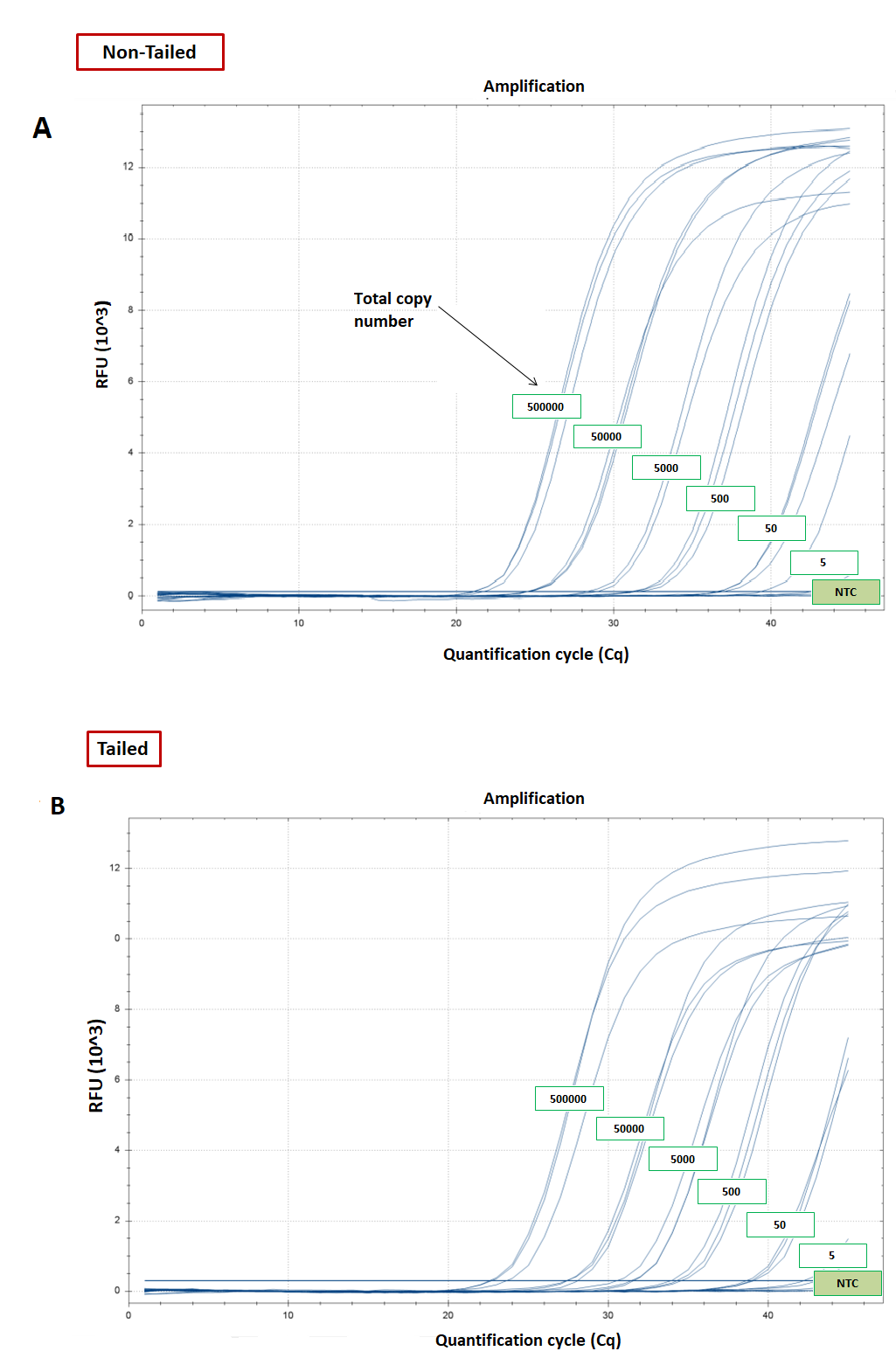


**S2** **Fig.** Amplification profiles of RdRp gene primers, when amplification is performed with A) non-tailed and tailed B) primers.

**S1** **Table.** List of Cq values removed from amplification profiles of genes E and RdRp.

**S3** **Fig.** Linear regressions of the plot of Cq as a function of the DNA copy number, for the case of E gene. The equations are shown both for the case of non-tailed and tailed primers.

**S2 Table.** Calculation of primer´s efficiency for E gene.

**S4 Fig.** Linear regressions of the plot of Cq as a function of the DNA copy number, for the case of RdRp gene. The equations are shown both for the case of non-tailed and tailed primers.

**S3 Table.** Calculation of primer´s efficiency for RdRp gene.

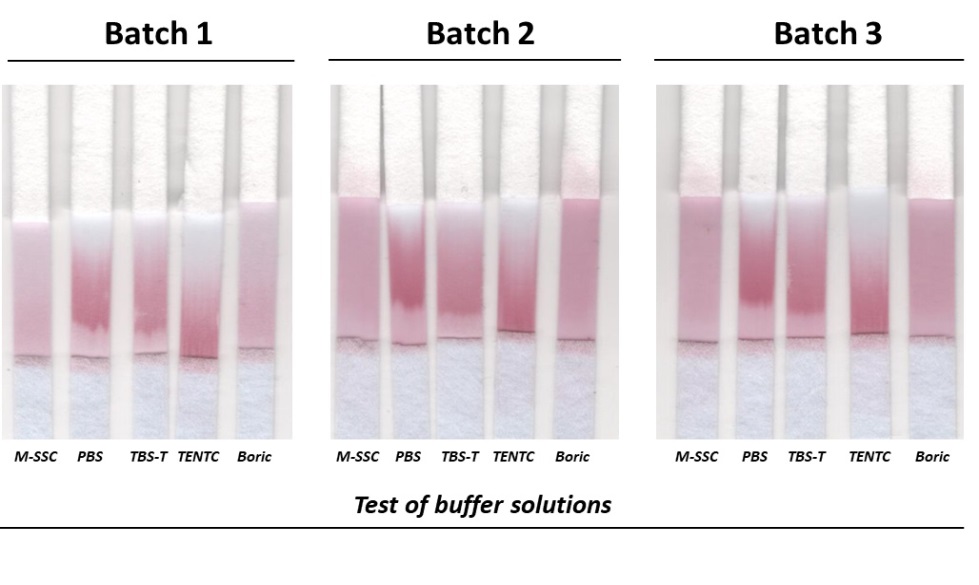


**S5 Fig.** Optimization of the running buffer to be used in the NALFA system; A total of 3 replicates/batches are exhibited.


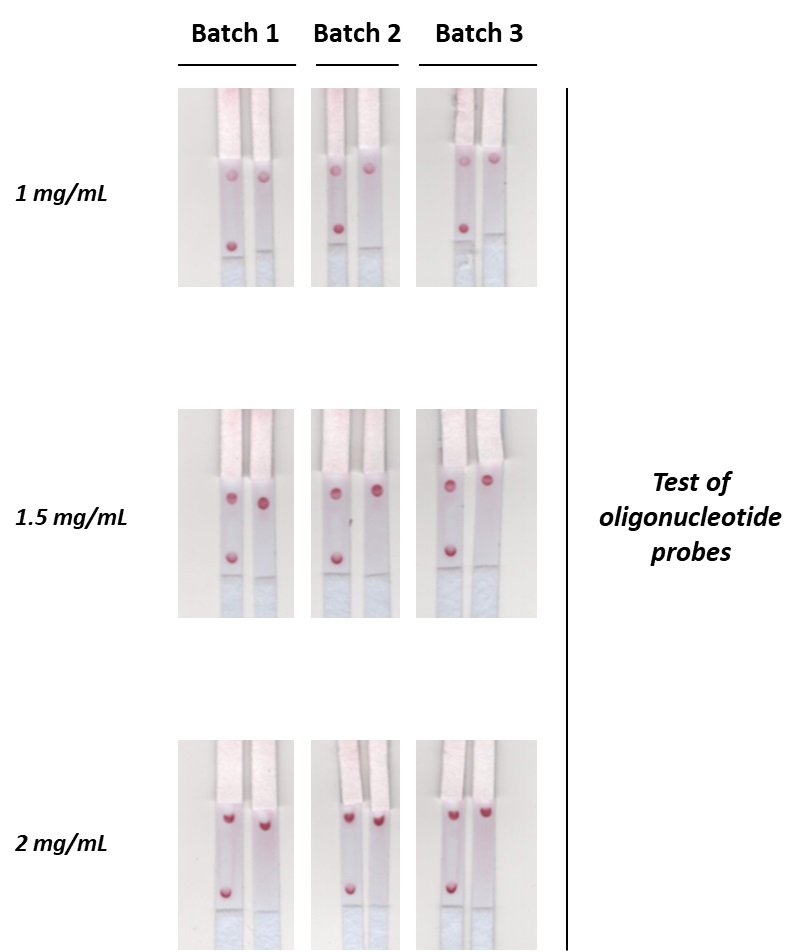


**S6 Fig.** Optimization of the capture oligonucleotide probes to be immobilized in the NALFA system; A total of 3 replicates/batches are exhibited.


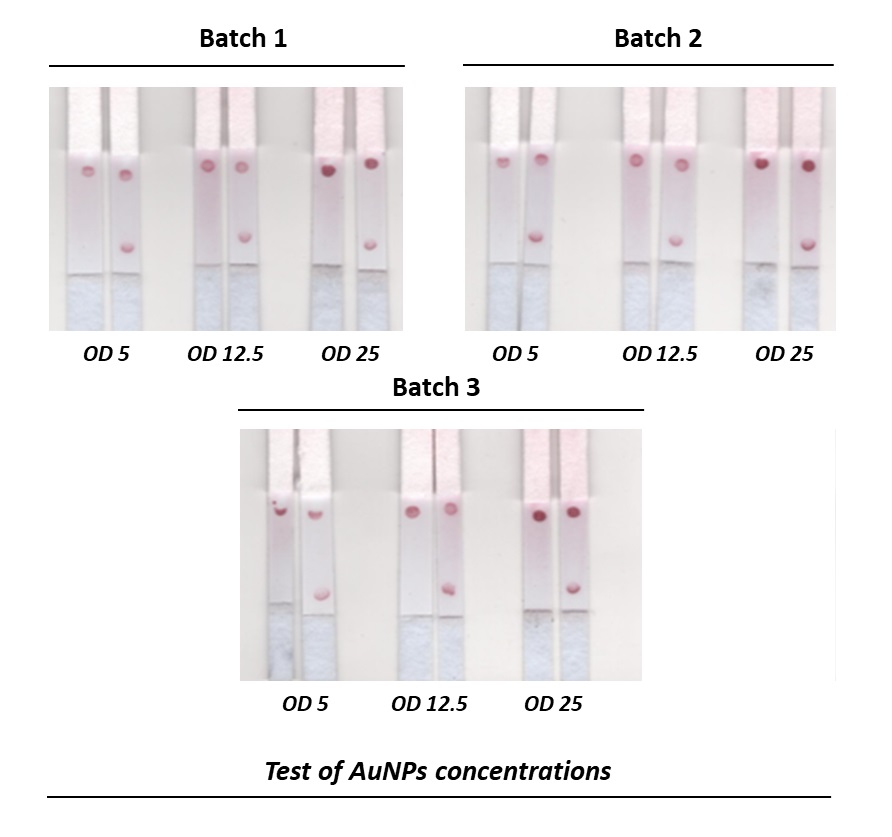


**S7 Fig.** Optimization of the AuNPs concentration to applied in the NALFA system; A total of 3 replicates/batches are exhibited.


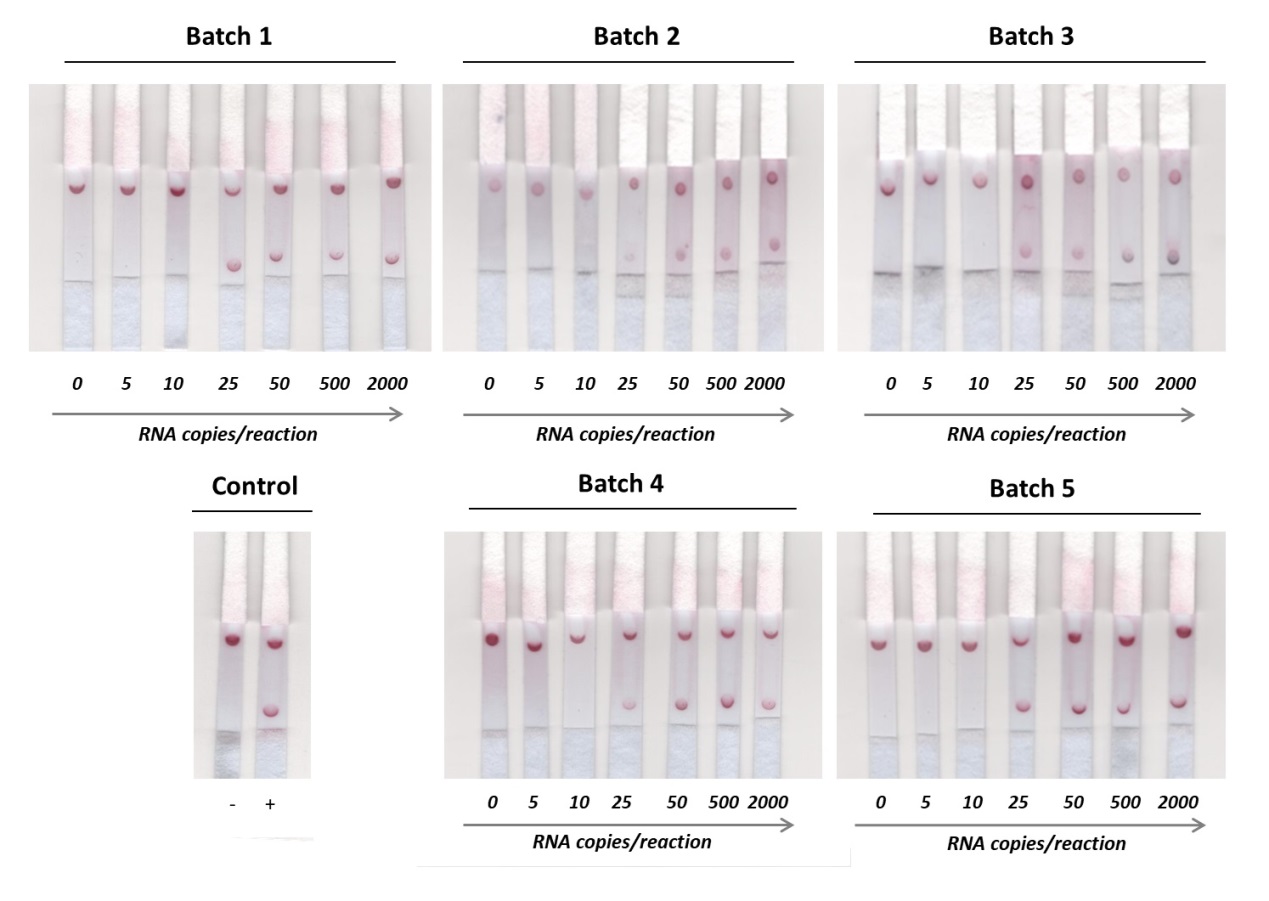


**S8 Fig.** Determination of the limit of detection (LOD) of the developed NALFA, using RNA as start point, expressed as the number of pre-amplification RNA copies, for the 5 replicates corresponding to the detection of E gene.


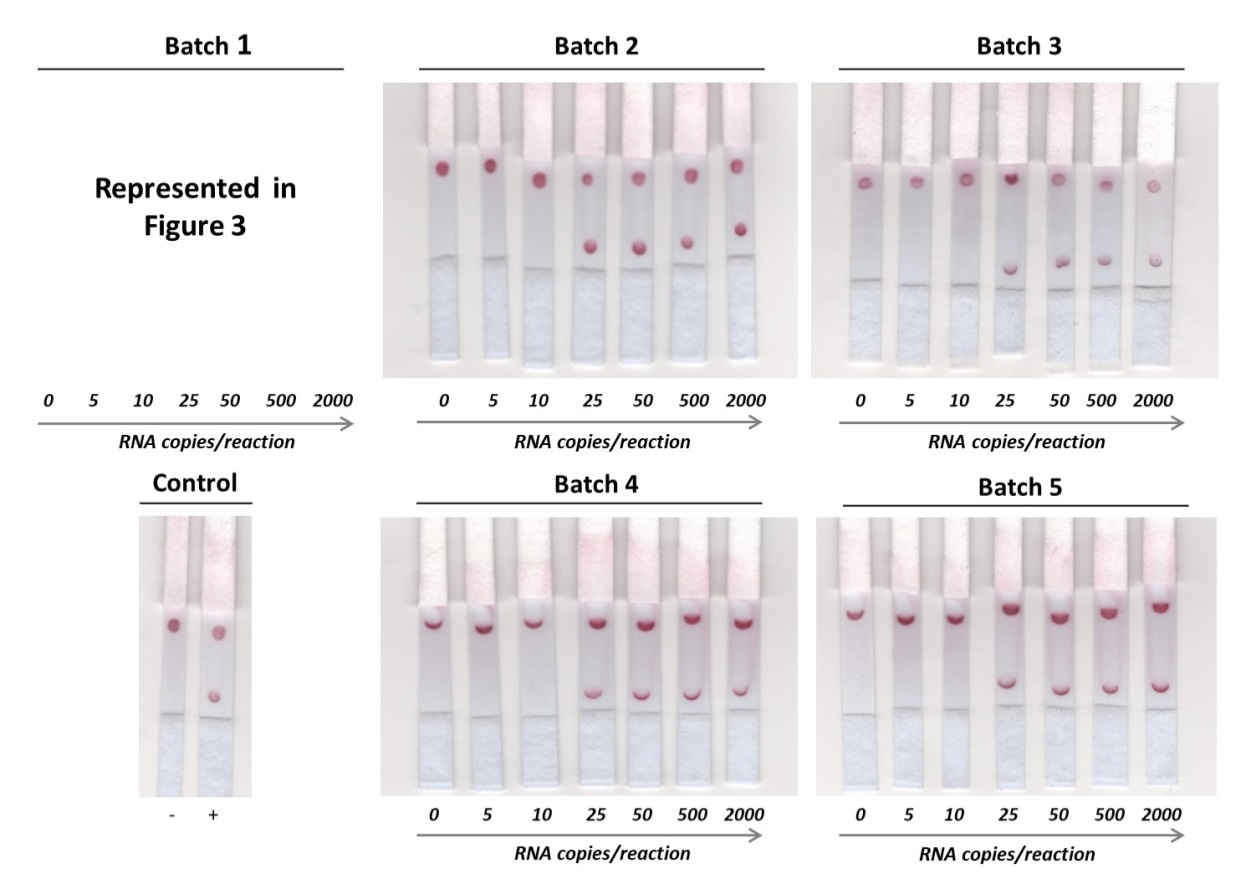


**S9 Fig.** Determination of the limit of detection (LOD) of the developed NALFA, using RNA as start point, expressed as the number of pre-amplification RNA copies, for the 4 out of 5 replicates corresponding to the detection of RdRp gene.


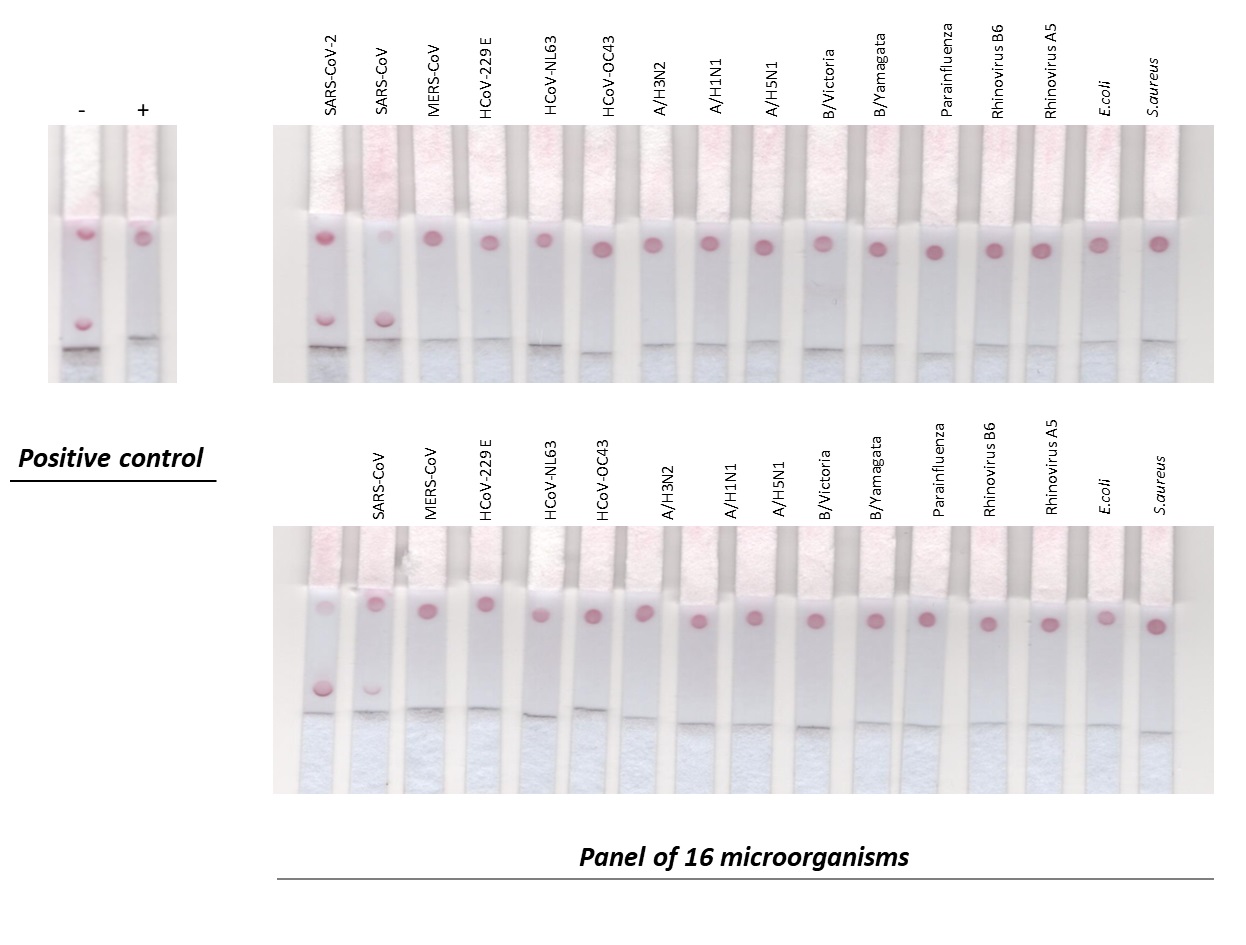


**S10 Fig.** Determination of the cross-reactivity of the developed NALFA, using a panel of distinct viruses and bacteria, for the case of E gene. Two replicates were executed.


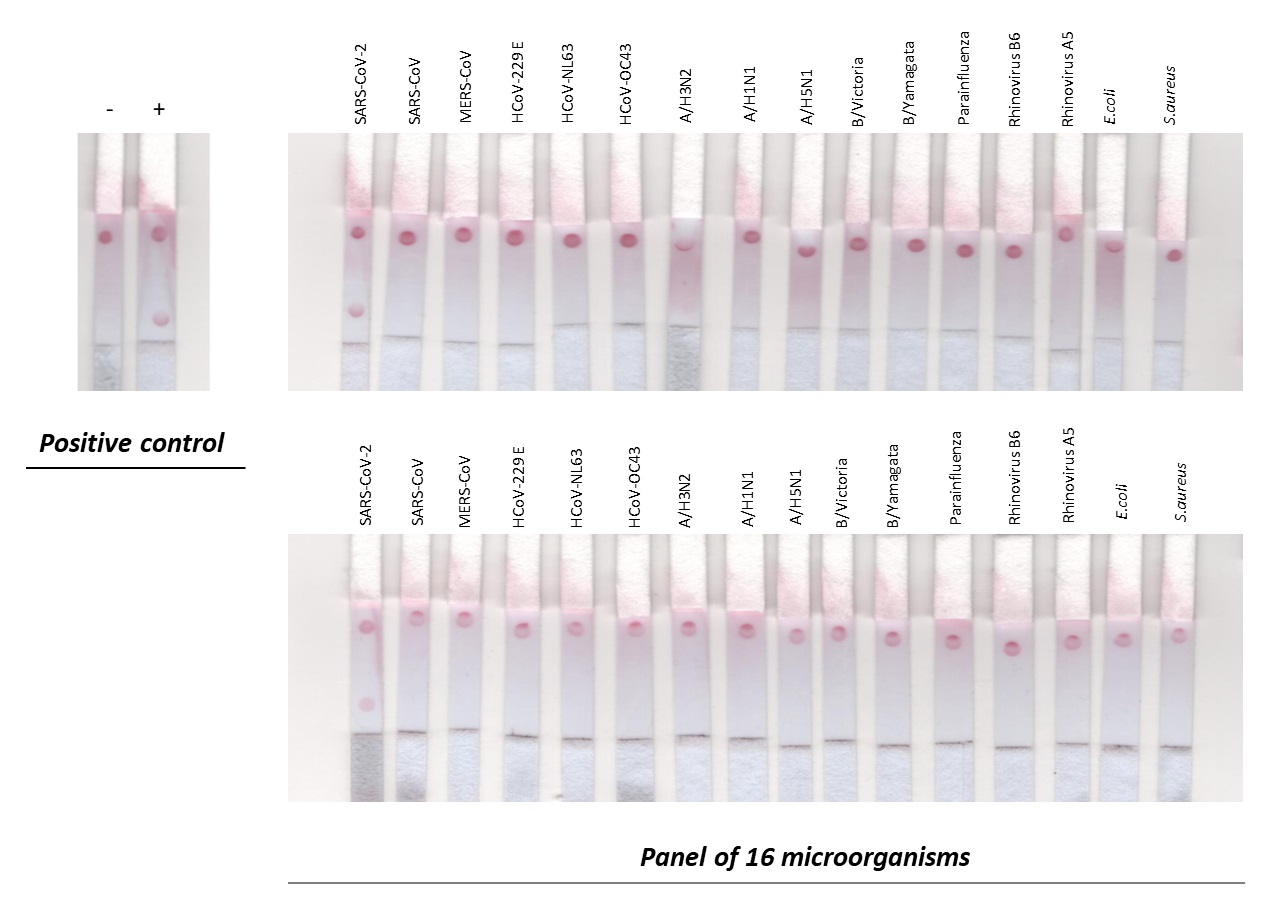


**S11 Fig.** Determination of the cross-reactivity of the developed NALFA, using a panel of distinct viruses and bacteria, for the case of RdRp gene. Two replicates were executed.


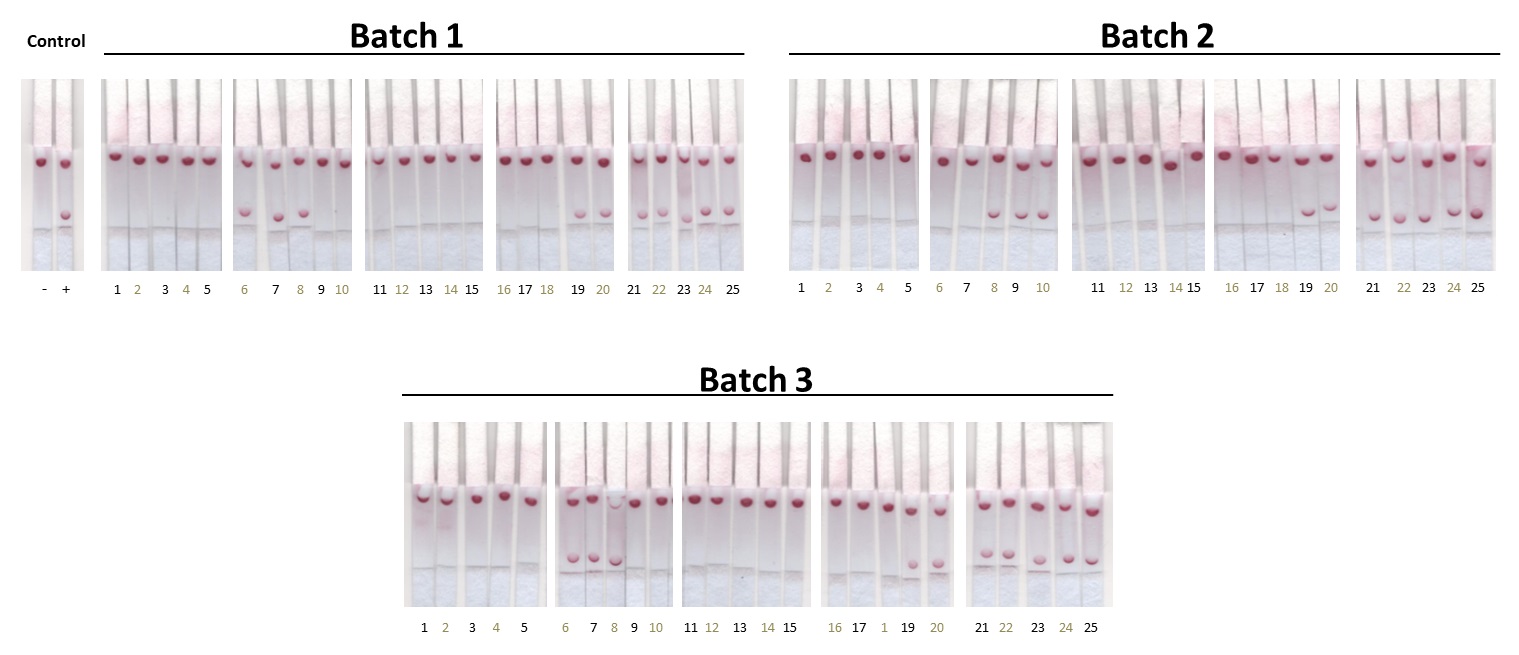


**S12 Fig.** Test of the 25 clinical specimens by NALFA, for the case of E gene detection (5 independent assays were performed, complementing the information in table 4 of the manuscript). Positive and negative results are indicated in the figure.


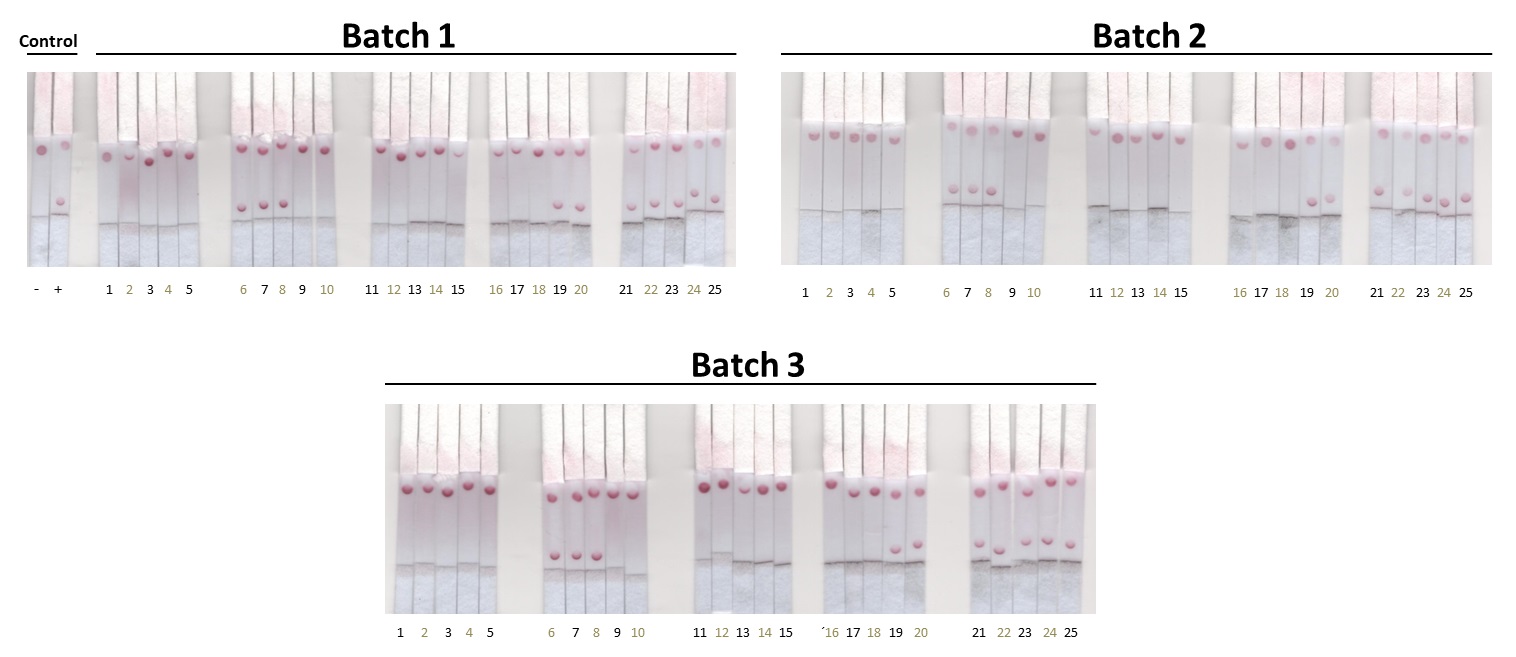


**S13** **Fig.** Test of the 20 clinical specimens by NALFA, for the case of RdRp gene detection (5 independent assays were performed, complementing the information in table 4 of the manuscript). Positive and negative results are indicated in the figure.
